# Biological traits of marine benthic invertebrates in Northwest Europe

**DOI:** 10.1038/s41597-022-01442-y

**Published:** 2022-06-15

**Authors:** David S. Clare, Stefan G. Bolam, Paul S. O. McIlwaine, Clement Garcia, Joanna M. Murray, Jacqueline D. Eggleton

**Affiliations:** Centre for Environment, Fisheries and Aquaculture Science, Pakefield Road, Lowestoft, UK

**Keywords:** Community ecology, Biodiversity

## Abstract

Biological traits analysis (BTA) provides insight into causes and consequences of biodiversity change that cannot be achieved using traditional taxonomic approaches. However, acquiring information on biological traits (i.e., the behavioural, morphological, and reproductive characteristics of taxa) can be extremely time-consuming, especially for large community datasets, thus hindering the successful application of BTA. Here, we present information on ten key biological traits for over a thousand marine benthic invertebrate taxa surveyed in Northwest Europe (mainly the UK shelf). Scores of 0 to 3 are provided to indicate our confidence that taxa exhibit each possible mode of trait expression. The information was acquired over a decade through an extensive appraisal of relevant sources, including peer-reviewed papers, books, online material and, where necessary, professional judgement. These data may be inspected, used, and augmented by fellow researchers, thus assisting in the wider application of BTA in marine benthic ecology.

## Abstract (summary)

**Table:** 

Measurement(s)	body size • body morphology • lifespan • egg development location • larva development location • living habit • sediment position • feeding mode • mobility • bioturbation mode
Technology Type(s)	literature review • professional judgement
Sample Characteristic - Organism	benthic invertebrates
Sample Characteristic - Environment	marine environment
Sample Characteristic - Location	Northwest Europe

## Background & Summary

Biological traits (i.e., morphological, behavioural, and life-history characteristics) determine how species respond to environmental variation and how they influence ecosystem functioning^1,2^. The study of biological traits can therefore be used to elucidate causes and consequences of biodiversity change that would go undetected using traditional analyses of community composition^3,4^. Biological traits analysis (BTA) originated in freshwater and terrestrial systems^5–7^ but is now frequently applied to marine systems, particularly the marine benthos^8–17^. Its application has advanced our understanding of human impacts on marine biodiversity and ecosystem functioning. With the seafloor globally subjected to increasing anthropogenic pressures^18^ and ecosystem functioning becoming ingrained within contemporary policy drivers for marine conservation (e.g., ecosystem-based fisheries management, the EU Marine Strategy Framework Directive), the number of benthic ecological studies applying BTA is likely to continue to rise.

A pre-requisite to BTA is acquiring the necessary biological trait information. Compiling information from primary sources (i.e., reports on laboratory or field observations) is time-consuming and often prohibitive given the large numbers of invertebrate taxa that typically comprise benthic assemblages. Secondary sources, such as online databases (e.g., BIOTIC http://www.marlin.ac.uk/biotic), provide repositories where compiled trait information can be readily accessed. However, extensive gaps have been highlighted in such databases regarding the basic biology of well-studied taxa, with up to 20% of demersal species surveyed in British marine waters completely lacking information for eight fundamental traits^19^. When information cannot be obtained from primary or secondary sources, trait expression can be inferred from the traits of closely related taxa. However, such inferences require knowledge on the biology of taxonomic groups and must be applied with caution when there is variation in trait expression across species from the same group.

This paper aims to assist researchers in sourcing biological trait information for large numbers of marine benthic invertebrate taxa. A freely accessible data matrix is provided, describing trait expression by 1,025 taxa (recorded at the genus level and above) for each of ten biological traits (Supplementary Table 1). This information has been acquired over a ten-year period, largely by benthic ecologists working at Cefas on projects with aims ranging from assessing the impacts of bottom trawling and aggregate dredging to mapping the functional characteristics of the benthos across broad-scale management boundaries. The provision of the resulting database augments other comparable initiatives (e.g., the *Polytraits* database^20^) and is intended to provide a reliable source of trait information to facilitate future application of BTA in marine benthic ecology.

## Methods

Biological traits information was obtained for the 1,025 taxa studied within projects undertaken by the authors of this paper. These projects focused on the ecology of sedimentary habitats in Northwest Europe, primarily UK shelf seas. Ten biological traits (maximum body size, morphology, longevity, egg development location, larval development location, living habit, sediment position, feeding mode, mobility, and bioturbation mode) were selected to address the ecological questions raised by the aims of these projects. Consequently, the ten traits cover a range of widely studied biological characteristics that determine how species respond to environmental variation and/or influence ecosystem functioning. Each trait was divided into between 3 and 6 categories, each representing a possible mode of trait expression or, for quantitative traits, a range of trait values (Supplementary Table 1).

Trait information was obtained using primary literature (i.e., papers and theses reporting on *in situ* observations and laboratory experiments^21–301^), secondary literature (i.e., textbooks and online databases^302–344^) and professional judgement (i.e., traits inferred from the traits of closely related taxa). Primary literature was given precedence over secondary literature when compiling trait information, and professional judgement was used only when information could not be obtained from the other two sources. Trait information was compiled at the genus level and above, accounting for the range of trait expression at lower taxonomic levels. For example, entries at the genus level captured any species-level variation in trait expression, while entries at the family level captured any variation in trait expression at the genus and species levels. Trait information was not compiled at the species level because members of the same genus tend to have consistent trait expression for the categorical traits used, and any apparent interspecific differences may equally be explained by all members of a genus having context-specific trait expression (discussed in the Usage Notes). As traits were assigned to taxa surveyed in Northwest Europe, trait information from sources pertaining to this region was prioritised.

To describe trait expression, taxa were given a numerical score for each category of all ten biological traits. The score ranged from 0 to 3, depending on the strength of the evidence that the taxon exhibits a trait category (0 = no evidence, 3 = strong evidence). For example, a taxon established as having planktotrophic larvae in the primary literature would be scored ‘3’ for ‘planktotrophic’, whereas a taxon for which there were unsubstantiated accounts of planktotrophic larval development in the secondary literature would be given a score of ‘2’. Scores of ‘1’ were given when professional judgement was required or when the literature suggests that trait expression by a taxon may vary across its constituent species. However, in cases where trait expression is identical among studied members of a taxonomic group (e.g., brooding of eggs by Amphipoda), members of the same taxonomic group for which direct evidence is lacking were assumed with confidence to also express the trait in the same way.

## Data Records

A matrix of biological traits information for 1,025 marine benthic invertebrate taxa, resulting from Methods described above, can be accessed online via the Cefas Data Portal at https://data.cefas.co.uk/view/21362^345^. Taxonomic nomenclature in this dataset was obtained from the World Register of Marine Species (WoRMS; http://www.marinespecies.org) on the 21^st^ of January 2022.

## Technical Validation

Trait information was compiled using the most reliable information available at the time of sourcing. A full list of sources used to compile trait information is provided in the References^21–344^. Outputs of analyses that used earlier versions of the accompanying trait matrix have been widely peer-reviewed and have improved our understanding of how benthic assemblages respond to anthropogenic pressures and influence ecosystem functioning^14–17,346–354^. The trait matrix has been augmented and refined over time as the list of taxa has expanded and new trait information has become available, culminating in a final review by authors in October 2021 to ensure that entries were consistent with our knowledge of the biology of the taxa. The identification of any entries as potentially erroneous was followed by a reinspection of the literature to resolve the uncertainty.

Due to the evolving nature of the trait matrix over the past decade and its various contributors, a limitation of the dataset is that a detailed account of the sources(s) of each entry (i.e., expression of an individual trait by an individual taxon) is not available. However, if users flag potentially erroneous entries with the corresponding author, then the literature will be reinspected, entries will be amended (if appropriate), and more detailed information on the sources of disputed entries will accompany future versions of the matrix. Moreover, it should be noted that despite primary and secondary sources being widely reviewed throughout the process of compiling trait information, many trait entries in the final matrix result from professional judgement. Such entries will also be amended in future iterations of the matrix if they are superseded by new information made available in the primary or secondary literature. Therefore, while the current matrix is intended to provide a useful and up-to-date resource for the scientific community, the process of refining this resource is ongoing.

## Usage Notes

The accompanying matrix does not cover an exhaustive list of ecologically important traits, but rather a subset of traits that are relevant to the ecological questions addressed by the authors of this paper under the auspices of several projects. We advocate that the choice of traits is carefully tailored to each application. Not all the ten traits will be appropriate for use in every study, while traits not included in the matrix may require consideration if they are relevant to the questions being addressed. Users should therefore draw on the information provided here only if it helps to achieve the specific aims of their research.

In compiling biological trait information, priority was given to trait expression by species in Northwest Europe, which is the region where the associated projects were conducted. Therefore, the information presented for a taxon in the accompanying matrix primarily reflects trait expression by species within this region. Species that occur only outside the region may express traits differently to congeners that occur only within the region, while populations of the same species located outside the region may also express traits differently. These possibilities should be considered if the information presented here is used to assign biological traits to taxa found outside of Northwest Europe.

Users of the accompanying matrix should consider the possible implications of taxa having multiple modes of expression (categories) for a single trait. This does not necessarily mean that each mode is equally likely in all environments, as trait expression may depend on abiotic or biotic context^355,356^. For example, a species may alter its feeding mode in response to anthropogenic disturbance^357^, hydrodynamic conditions^358^, water temperature and chemistry^282^ or interspecific interactions^359^. Reliable information on the conditions in which taxa express traits in specific ways is rarely available and was therefore not incorporated into the accompanying trait matrix. This may affect the reliability of BTA outputs, depending on the ecological questions addressed.

Finally, we reiterate that the associated trait matrix reflects the best information available to the authors at the time this paper was submitted. The matrix will continue to be augmented and refined over time as the taxon list grows and new information about trait expression becomes available. It is our intention to make updated versions accessible through the Cefas Data Portal (https://data.cefas.co.uk) and for these future versions to be ‘signposted’ on the page where the current matrix can be accessed (https://data.cefas.co.uk/view/21362). We therefore encourage users to check, verify and, if necessary, amend the trait information provided here prior to use. Other researchers working independently of the authors of this paper have compiled and published trait information for marine benthic invertebrates, focussing on specific taxonomic groups^20^ or traits that influence specific ecological processes^360^. We advise that users crosscheck between sources and review the primary literature to attempt to resolve any areas of disagreement. We also advise that users are particularly cautious when using information from the accompanying matrix that has a confidence score of ‘1’. This low score should be taken as a prompt to review the literature for information that may have been made available since the publication of this paper and may be used to direct future empirical research on the traits of marine benthic invertebrates. Any evidence that corroborates or contradicts information in the trait matrix provided here, particularly that which is derived from empirical research, is welcomed via email to the corresponding author. This evidence will be incorporated into future versions of the trait matrix.

## Supplementary information

Supplementary Table 1

## Data Availability

No custom code was used to generate or process the data described in the manuscript.
