# Supplementary material for: Biological traits of marine benthic invertebrates in Northwest Europe
Source: Sci Data. 2022 Jun 15;9:339. doi: 10.1038/s41597-022-01442-y (PMC9200785; doi:10.1038/s41597-022-01442-y)
Supplement: Supplementary file 1 — Supplementary Table 1 [file 41597_2022_1442_MOESM1_ESM.docx]

Supplementary Table 1. Biological traits used to describe benthic invertebrate taxa. Trait abbreviations used in the data matrix are in parentheses.

| **Trait** | **Category** | **Description** |
| --- | --- | --- |
| Maximum size (sr) | < 10 |  |
|  | 10–20 |  |
|  | 21–100 | The maximum size (mm) that the taxon is known to reach during the adult stage. |
|  | 101–200 |  |
|  | > 200 |  |
| Morphology  (m) | Soft | External tissue is soft and not covered by any form of protective casing. |
|  | Tunic | Body is covered by a protective outer tissue made up of, for example, cellulose, e.g., tunicates. |
|  | Exoskeleton | Body is covered or encased in either a thin chitinous layer or calcium carbonate shell. |
|  | Crustose | Body is hard and forms a thin layer over the substratum or another organism. |
|  | Cushion | Body is soft and forms a cushion-like layer over the substratum or another organism. |
|  | Stalked | Body is erect and typically attached. |
| Lifespan  (l) | < 1 |  |
|  | 1–3 | The maximum reported lifespan (years) of the adult stage of the taxon. |
|  | 3–10 |  |
|  | > 10 |  |
| Egg development location  (ed) | Asexual | Can reproduce asexually, either by fragmentation, budding, epitoky, etc. |
|  | Sexual - pelagic | Eggs are released into the water column. |
|  | Sexual - benthic | Eggs are released onto/into the seabed, either free or attached, e.g. by mucous. |
|  | Sexual - brooded | Fertilised eggs are maintained by adult for protection, either within parental tube or body cavity. |
| Larval development location  (ld) | Pelagic - planktotrophic | Larvae feed on plankton and spend a relatively long time in the water column. |
|  | Pelagic - lecithotrophic | Larvae feed on yolk reserves and spend a relatively short time in the water column. |
|  | Benthic (direct) | Larval stage missing (eggs develop directly into juveniles) or larvae are limited to the seabed. |
| Living habit  (lh) | Tube-dwelling | Adults live in a tube, which may be lined with sand, mucus or calcium carbonate. |
|  | Burrow-dwelling | Adults live in a permanent or temporary burrow. |
|  | Free-living | Adults do not inhabit a restrictive structure. Able to move freely within and/or on sediments. |
|  | Crevice/hole/under stones | Adults typically cryptic and inhabit spaces within coarse/rock substrate or algal holdfasts. |
|  | Epi/endo-biotic | Adults live on or in another organism. |
|  | Attached to substratum | Adults are attached to coarse substrate or rock. |
| Sediment position  (sp) | Surface | Adults live on or just above the seabed. |
|  | Shallow infauna | Adults live below sediment surface between 0 and 5 cm depth. |
|  | Mid-depth infauna | Adults live below sediment surface between 5 and 10 cm depth. |
|  | Deep infauna | Adults live below sediment surface at greater than 10 cm depth. |
| Feeding mode  (f) | Suspension | Feeds on particulate food resources suspended in the water column. |
|  | Surface deposit | Feeds on detritus (including algal material) on the sediment surface. |
|  | Sub-surface deposit | Feeds on detritus located within the sediment matrix. |
|  | Scavenger | Feeds on dead animals (carrion). |
|  | Predator | Actively predates on animals (including small zooplankton). |
|  | Parasite | Derives nutrition from its host organism. |
| Mobility  (mob) | Sessile | Adults have little or no mobility. Typically attached or lives in (semi-) permanent burrow/tube. |
|  | Swim | Adults actively swim in the water column (many return to the bed when not feeding). |
|  | Crawl/creep/climb | Adults capable of some (typically limited) movement along the sediment or rock surface. |
|  | Burrower | Adults capable of active movement within the sediment matrix. |
| Bioturbation mode  (b) | Diffusive mixing | Vertical and horizontal redistribution of sediment and/or other particles. |
|  | Surface deposition | Deposition of particles at sediment surface, e.g. from defecation or egestion (pseudofaeces). |
|  | Upward conveyor | Translocation of particles from depth to sediment surface, e.g. during sub-surface deposit-feeding. |
|  | Downward conveyor | The subduction of particles from sediment surface to depth, e.g. by feeding and/or defecation. |
|  | None | Does not have any bioturbative capacity. |
